# New-onset of pemphigus following COVID-19 infection: A case report

**DOI:** 10.1177/2050313X241231423

**Published:** 2024-02-16

**Authors:** Elena Pastukhova, Feras M Ghazawi

**Affiliations:** 1 Department of Medicine, University of Toronto, Toronto, ON, Canada; 2 Division of Dermatology, The Ottawa Hospital, University of Ottawa, Ottawa, ON, Canada

**Keywords:** pemphigus, pemphigus vulgaris, paraneoplastic pemphigus, autoimmune bullous disease, SARS-CoV-2, COVID-19, immunology

## Abstract

COVID-19 has been implicated in various cutaneous autoimmune diseases. Pemphigus is a group of autoimmune blistering diseases that target the desmosomal complexes. Pemphigus triggered by COVID-19 has been seldom reported in the literature and remains both a diagnostic and therapeutic challenge. We report a case of COVID-19-induced pemphigus that responded well to prednisone and mycophenolate mofetil after 9 months from initial presentation. On histologic examination, both intercellular and basement membrane staining were noted. Indirect immunofluorescence staining was positive against the intercellular cement of the stratified epithelium from monkey esophagus. We hypothesize that COVID-19 stimulated the release of multiple pemphigus antigens, which resulted in the unusual histologic pattern reported in the present case. Although malignancy should be suspected when features of paraneoplastic pemphigus, such as basement membrane staining on direct immunofluorescence, are noted, it may also be a histologic pattern of pemphigus secondary to COVID-19 that clinicians may consider.

## Introduction

Pemphigus is a heterogeneous group of mucocutaneous autoimmune bullous diseases resulting from antibody-mediated acantholysis. Pemphigus subtypes include pemphigus vulgaris (PV), pemphigus foliaceus, pemphigus vegetans, pemphigus herpetiformis, IgA pemphigus, and paraneoplastic pemphigus (PNP). PV is the most common subtype, accounting for 60%–90% of all cases.^1^ The antigenic targets of pemphigus disease are desmosomal complexes, such as desmoglein and desmocollin, which dictate the clinical presentation and disease subtype. While most subtypes of pemphigus disease typically manifest as blisters and erosions, PNP may mimic other dermatoses morphologically. Unlike other pemphigus subtypes, PNP generally carries an unfavorable prognosis and a high mortality rate.^1^

Multiple autoimmune diseases have been shown to be triggered by COVID-19.^2^ Despite some reports,^3^ there is limited data on the incidence of pemphigus following COVID-19 infection. Herein, we present a case of a 73-year-old female with new-onset pemphigus 2 weeks after COVID-19 infection.

## Case report

**Figure 1.**
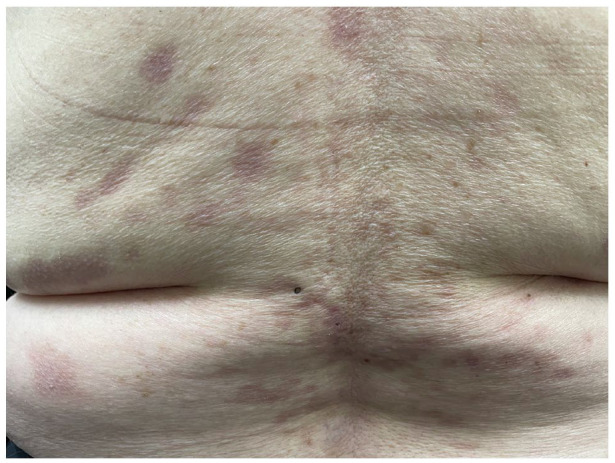
On the posterior trunk, there are numerous pink-brown, post-inflammatory macules and patches, ranging from 0.5 to 2 cm in size, with no active bullae.

**Figure 2.**
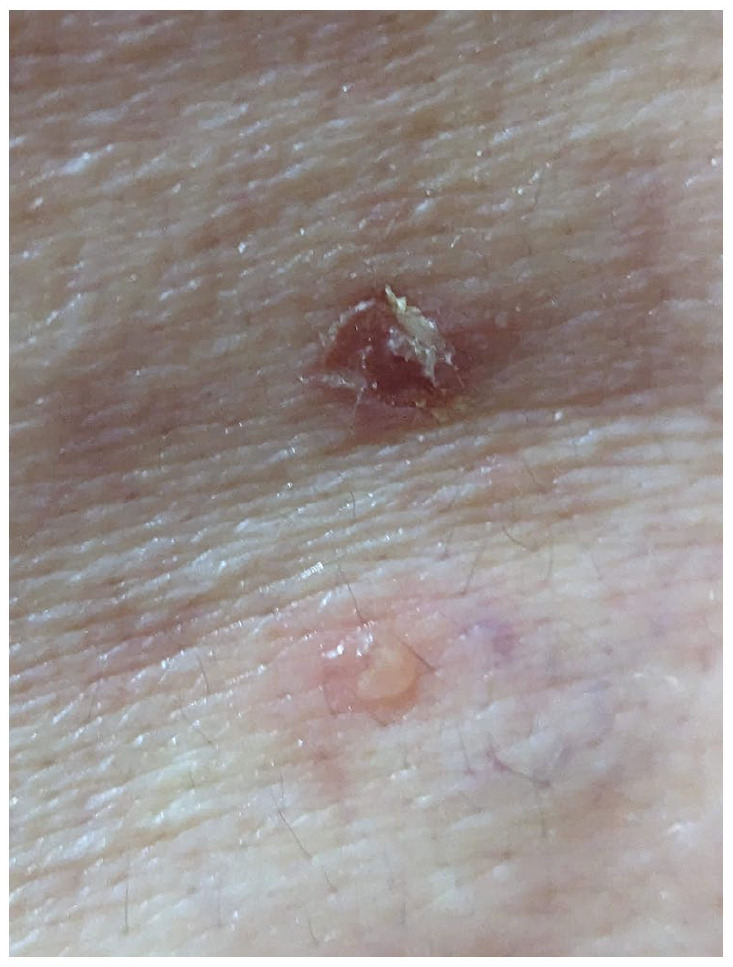
On the posterior trunk, there is a 0.5 cm erosion above a 0.3 cm vesicle.

A 73-year-old female with Fitzpatrick II skin presents for evaluation of a blistering eruption of her neck, bilateral axillae and arms, anterior and posterior torso, and genital region that started 2 weeks after testing positive for COVID-19 via rapid antigen testing. Review of systems was otherwise unremarkable, including for malignancy. She has no previous dermatologic or autoimmune history. She denies starting any new medications within the past year. On examination, there are numerous erosions, vesicles and bullae, and pink-brown, post-inflammatory macules and patches on her anterior and posterior trunk, ranging from 0.5 to 2 cm in size (Figures 1 and 2). No history or evidence of mucosal involvement or lymphadenopathy was noted. Punch biopsies of lesional skin demonstrated mild dermal scarring and mild acanthosis. Direct immunofluorescence of perilesional skin showed intercellular space and cell surface staining patterns. IgG showed strong staining, while C3 showed weaker staining that was enhanced along the basement membrane. Indirect immunofluorescence staining for IgG antibodies against the intercellular cement of the stratified epithelium from monkey esophagus was present. These features were most compatible with PV. However, note was made of the enhanced basement membrane staining suggestive of PNP. As such, a thorough malignancy work-up was completed, including a thyroid ultrasound, full body CT imaging, mammogram, and fecal occult blood test, which were unremarkable for malignancy. C-reactive protein was 16.4, anti-nuclear antibody was negative, and anti-skin antibody was positive (1:80) with a pemphigus pattern. Serum protein electrophoresis with immunofixation was within normal limits. At 9 months of follow-up and thorough testing, no evidence of malignancy was found.

Patient was initially prescribed a course of prednisone 30 mg with a taper, with some response. She was subsequently started on mycophenolate mofetil, following which she had a complete and sustained remission at 9 months of follow-up.

## Discussion

Viruses have long been recognized as triggers of autoantibody production and multiple mediated autoimmune diseases, including pemphigus.^4^ Similarly, SARS-CoV-2 has been implicated in etiopathogenesis of IgG-mediated autoimmunity.^2^ Several theories have been proposed as to the mechanisms of such. Studies have previously characterized homology between the human proteome and SARS-CoV-2 viral elements.^2^ As such, cross-reactivity may occur, leading to autoantibodies production in susceptible individuals. This molecular mimicry is further facilitated by the ability of SARS-CoV-2 to hyper-stimulate the immune system, leading to the production of many pro-inflammatory cytokines and higher potential for autoimmune pathogenesis.

Several reports have described de novo PV following COVID-19 infection and vaccination.^3^ One study described new onset of PV 40 days following COVID-19 infection.^5^ Moreover, the levels of anti-SARS-CoV-2 IgG in serum and saliva have been shown to peak around days 16–30 after symptom onset and remain stable for 105 days.^6^ The total IgG levels were also significantly higher in patients with COVID-19 when compared to healthy controls (*p* < 0.0001). It is plausible that through molecular mimicry, COVID-19 elicited an aberrant IgG-mediated immune response to desmosomal proteins. This could explain the time course of PV onset observed in our patient, as well as the previous case reports.^3,5^

The serum cytokine profile of PV demonstrates significantly lower levels of interferon (IFN) in PV when compared to controls, suggesting against an increased Th1 response often noted in autoimmune disease.^7^ IFN has been studied as a potential trigger of PV, with one study demonstrating new-onset PV after exogenous IFN therapy.^8^ COVID-19 is known to induce high levels of IFN.^9^ As such, it is possible that the increased levels of circulating IFN secondary to COVID-19 infection alter the homeostasis in the Th1/Th2 pathways, leading to increased Th2 signaling, self-B-lymphocyte activation, and subsequent autoimmunity.

Interestingly, our patient presents with new-onset pemphigus that is histologically consistent with PV but was notable for PNP features, which has not been reported previously. Unlike other pemphigus subtypes, PNP may induce autoimmunity to a diverse variety of desmosomal and cytoskeletal antigens, such as plakins and desmocollins.^1^ COVID-19 infection may have thus stimulated the production of multiple self-antigens and subsequent autoimmune IgGs,^2^ which ultimately mimicked PNP histologically with the enhanced basement membrane deposition. Furthermore, a cell-mediated response has been implicated in PNP, whereby both activated CD8+ T-cells and natural killer (NK) cells have been located at the dermo-epidermal junction.^10^ COVID-19 garners a strong CD8+ T-cell and NK cytotoxic response even after acute infection.^9^ Thus, the basement membrane staining pattern observed in the present case may reflect COVID-19-induced activation of the junctional immune microenvironment. Our report presents a novel diagnostic challenge, whereby a history of COVID-19 infection may be considered in addition to a thorough malignancy work-up in pemphigus etiopathogenesis.
